# Quality Management of Pulmonary Nodule Radiology Reports Based on Natural Language Processing

**DOI:** 10.3390/bioengineering9060244

**Published:** 2022-06-01

**Authors:** Xiaolu Fei, Pengyu Chen, Lan Wei, Yue Huang, Yi Xin, Jia Li

**Affiliations:** 1 Information Center, Xuanwu Hospital, Capital Medical University, Beijing 100053, China; feixiaolu@xwh.ccmu.edu.cn (X.F.); pengmc_ce@163.com (P.C.); weilan@xwhosp.org (L.W.); huangyue@xwhosp.org (Y.H.); 2 School of Life Science, Beijing Institute of Technology, Beijing 100081,China; ameko@bit.edu.cn

**Keywords:** natural language processing, radiology report, quality management, knowledge graph, pulmonary nodule

## Abstract

To investigate the feasibility of automated follow-up recommendations based on findings in radiology reports, this paper proposed a Natural Language Processing model specific for Pulmonary Nodule Radiology Reports. Unstructured findings used to describe pulmonary nodules in 48,091 radiology reports were processed in this study. We established an NLP model to extract information entities from findings of radiology reports, using deep learning and conditional random-field algorithms. Subsequently, we constructed a knowledge graph comprising 168 entities and four relationships, based on the export recommendations of the internationally renowned Fleischner Society for pulmonary nodules. These were employed in combination with rule templates to automatically generate follow-up recommendations. The automatically generated recommendations were then compared to the impression part of the reports to evaluate the matching rate of proper follow ups in the current situation. The NLP model identified eight types of entities with a recognition accuracy of up to 94.22%. A total of 43,898 out of 48,091 clinical reports were judged to contain appropriate follow-up recommendations, corresponding to the matching rate of 91.28%. The results show that NLP can be used on Chinese radiology reports to extract structured information at the content level, thereby realizing the prompt and intelligent follow-up suggestion generation or post-quality management of follow-up recommendations.

## 1. Introduction

Radiology reporting is the final and crucial step of the computed tomography (CT) radiology exam, and follow-up suggestions in its “Impression” part always present a direct link of communication between clinicians and patients. Quality management of the radiology reports is conventionally required to ensure the “Impression” of report is consistent with the CT images and proper follow-up suggestions are presented in the report to avoid over- or under-medicating.

Quality management approaches, based on manual intervention process and departmental systems, did aid in the reduction in errors in reports [1,2,3]. To improve the overall quality of the radiology reports, official guidelines from various associations provide the templates and standards. However, because the quality of the report relies highly on the knowledge and experience of the individual senior radiologist reviewer, it is difficult to establish a consistent quality management model even within a healthcare organization [4,5]. Management methods relying on manual intervention cannot provide consistent and objective process specifications. The lack of consistent and stable quality management methods in radiology reports may lead to the lack of strict follow-up suggestions, which leads to unnecessary imaging examinations and the overuse of medical insurance resources. Many radiology experts agree that structured radiology reports are more complete and more effective than unstructured reports [6,7], which can provide a good foundation for automatic quality control based on the content of reports. However, in China, unstructured reports are still widely used in healthcare organizations so far, which poses credible challenges to automatic quality management. 

Structured reports can not only improve the consistency and clarity of reports, but also assist radiologists to extract information and in the subsequent clinical decision making [8,9,10]. Furthermore, in-depth and accurate information must undergo post-processing to enable assessment of the quality of the report in multiple aspects. Therefore, natural language processing (NLP) techniques are increasingly applied in various fields and languages [11,12,13]. Gershanik et al. performed NLP of chest CT reports to determine consistency in the detection of pulmonary nodules [14]. Duszak Jr. et al. employed NLP to determine whether radiologists’ records in abdominal ultrasound reports are sufficient for diagnosis and to subsequently evaluate the report quality and its influence on medical costs [15]. The American College of Radiology (ACR) developed a lung imaging reporting and data system (Lung-RADS) as a quality assurance tool for standardized lung cancer screening, reporting, and management recommendations [16]. The tools embedded in medical information systems are increasingly employed in clinical applications, such as learning tools to aid in the training of radiologists [17,18,19]. Nobel et al. used natural language processing technology to process radiological reports in Dutch for automatic classification of lung tumors [20]. S. Pathak et al. post-structured the Dutch radiological free text report by taking the TF-IDF value of each word and the length of each sentence as the characteristics [21]. However, there are few studies on post-structured reports and automatic quality management in Chinese radiology reports currently.

In recent years, Transformer has been used as main algorithm in NLP. Agnikula Kshatriya et al. used BERT and Bio-BERT to extract the text features of electronic health records and used semi-supervised training methods to evaluate doctors’ documents [22]. Hyun Gi Lee et al. used BERT and LSTM to extract the depth features of radiological reports [23]. Sanjeev Kumar Karn et al. designed a word-level encoder and a sentence-level encoder based on BI-LSTM to automatically generate a radiological report summary [24]. Song Wang et al. extracted information nodes from radiology reports and constructed knowledge maps to assist the generation of reports [25]. Arnaud et al. used BERT to learn text representation from French free text note and check the quality of the learned embedding based on a set of clustering models [26]. 

In this study, we presented the intelligent quality management method in a Chinese radiology report for the first time. BI-LSTM + CRF were adopted to extract the entities of Chinese radiological reports. The key information entities associated with follow-up recommendations for pulmonary nodules were identified, and a follow-up suggestion was rendered based on a knowledge graph and rule template. Some unique problems of the Chinese corpus were overcome in the implementation process, such as the shortage of registered word vectors of Chinese medical terms and the lack of an available authoritative specialized medical knowledge database. Through comparing the rendered follow-up suggestion with the text in the “Impression” part of the original report, the feasibility of automatic quality management method is investigated and verified, which could be helpful to decreasing the work load of subjective imaging report quality management and increasing the radiology report quality in Chinese.

## 2. Materials and Methods

### 2.1. Datasets

This retrospective study considered CT imaging of pulmonary nodules as the specific example of a medical application. The content of the radiology report extracted in this study is a summary of the description of routine diagnosis and treatment and does not involve any patient’s personal information. Chest CT radiology reports (N = 115,754) from a tertiary hospital in Beijing were collected between January 2015 and June 2019. All patients provided informed consent regarding the use of their data. Only the radiology reports involving pulmonary nodule descriptions and diagnoses were retained after screening (N = 52,089, 45%). Subsequently, the content with pulmonary nodule descriptions was filtered and cleaned, and false and redundant text data were removed, such that all included data were correct, objective, and uniform in format (N = 52,035; 45%). Subsequently, the reports of outpatients and inpatients (who were not subjected to a physical examination) with a history of cancer or immunodeficiency and those who were not suitable for lung cancer screening were excluded. Finally, N = 48,091 (42%) radiology reports were included in the study.

These data were input in both the NLP model and the follow-up generation model. In the NLP model, the 48,091 reports were randomly divided into three sets, namely a training set (N = 24,046; 50%), a verification set (N = 12,023; 25%), and a test set (N = 12,022; 25%) while ensuring that the patients’ age and anomaly distributions of each subset were consistent with those of the entire dataset.

**Figure 1:**
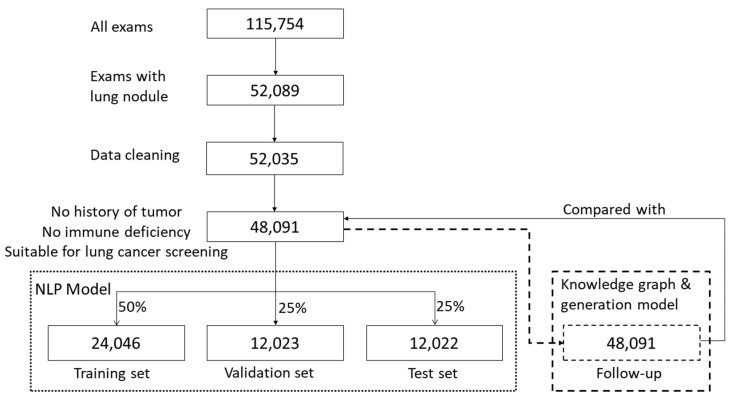
Flowchart of dataset processing.

In the follow-up generation model, 48,091 reports processed by the NLP model generated follow-up recommendations that were subsequently compared with those of the original reports to evaluate the quality management. Figure 1 summarizes the dataset processing workflow.

### 2.2. Methods

**Figure 2:**
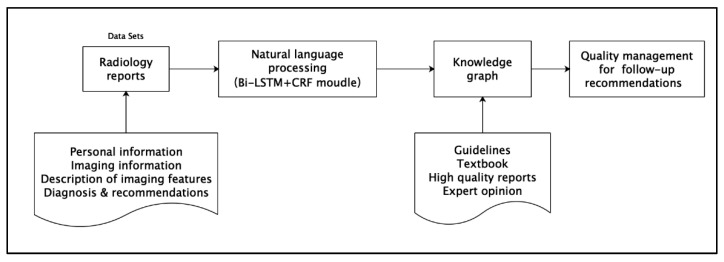
Technical roadmap for pulmonary nodule follow-up recommendations.

The method employed in this study comprises three steps, and the technical roadmap is presented in Figure 2.

#### 2.2.1. NLP Model

The bi-directional long short-term memory + conditional random field (Bi-LSTM + CRF) model was employed for NLP during the implementation stage. Pulmonary nodule descriptions in radiology reports were manually labeled and categorized into two groups. The first included descriptions displayed in the image, such as the location, size, number, shape, solidity and risk level, whereas the second included image diagnoses, as well as diagnoses and follow-up recommendations regarding pulmonary nodules detected in the report. All manually labeled content was provided by radiologists with many years of radiology work experience who underwent specialized training at the tertiary hospital in Beijing. There was no overlap between the doctors participating in the labeling work and those who established the labeling standards to ensure unbiased comparison of results.

The experiments are implemented in Anaconda + PyCharm with Python3.6, using Keras [27] library with a Tensorflow [28] backend. 

#### 2.2.2. Knowledge Graph Design

During the construction and implementation of the knowledge graph, the following guidelines were adopted: Fleischner Society’s authoritative guidelines on pulmonary nodule follow-up recommendations [29,30]; the eight sets of medical textbooks of the Ministry of Health of the People’s Republic of China; Chinese experts’ guidelines on the classification, diagnosis, and treatment of pulmonary nodules [31,32,33]; ACR guidelines on Lung-RADS imaging reporting and data system [15,16], and high-quality radiology reports. During the mapping between the English guidelines and Chinese radiology reports, the Chinese version of SNOMED was employed, as it defines clinical concepts with unique identifiers and descriptive features.

To ensure coverage of all information query results, it is critical to correctly set the entities and their relationships in the knowledge graph. We assigned six types of entities, including nodule name, size, solidity, quantity, risk level and follow-up recommendations, as well as four relationships, including “has,” “has_an_attribute,” “follow_up_is”, and “same_as”.

Based on these entities and relationships, we compiled a knowledge graph of pulmonary nodule follow-up recommendations, according to descriptions in authoritative guidelines, medical textbooks and high-quality reports. The Neo4j graph database, regarded as a high-performance graph engine with all the features of a mature database, was adopted as the knowledge graph tool during the implementation, owing to its advantages of embedment, high performance, and light weight.

#### 2.2.3. Generation of Intelligent Follow-Up Recommendations

The rule-based reasoning template was employed to locate a pulmonary nodule’s status in the knowledge graph through the slot value and relationships of NLP. On this basis, we applied the WHERE clause in the MATCH command to filter the results of the MATCH query to derive follow-up recommendations. Five variables, namely a, b, c, d and e that represent the name, solidity characteristics, size, quantity and risk level of the nodule, respectively, were defined. Subsequently, slot values were filled into the variables according to the NLP results. Finally, follow-up recommendations were generated based on the attribute values of entities and relationships. If the output and text comparison results were consistent with frequency and follow-up methods, then the comparison was considered consistent.

## 3. Results

A total of 48,091 reports were used in this research. Because the system integrated different modules, the results are presented in the following three sections: NLP model results, knowledge graph construction and demonstration of the entire system.

### 3.1. NLP Model Results

Seven practical attributes of the nodule and one follow-up attribute were acquired after processing. The number of entities with different attributes and model evaluation indicators are listed in Table 1, which presents the results of the best-performing feature extraction. The overall accuracy, precision, recall and F1 of the NLP model are 94.22%, 94.56%, 93.96% and 94.26%, respectively. Notably, the NLP model performed well in terms of expressing relatively fixed entity types. The accuracy and precision of location (96.71%, 96.00%), shape (94.62%, 96.70%) and nodule name (97.21%, 98.95%) are higher than the overall level. Due to individual variations in the nodule sizes, the performance indicators for size recognition perform lower than the overall level. These higher or lower levels are not statistically different.

There are 97 entities after standardization, which serve as an objective manifestation of various methods employed by doctors to describe the pulmonary nodule entity. There are 37, 11, 13, 7, 6, 14 and 9 entities regarding the location, shape, nodule name, solidity, quantity, risk level and follow-up recommendation, respectively. The nodule size is meaningless for the calculation of the entity number of pulmonary nodules due to its large variability. Therefore, the corresponding “Number” cell was not counted, whereas other evaluation indicators of the model describing the size remained incorporated in the performance evaluation.

### 3.2. Graph Database Construction

**Figure 3:**
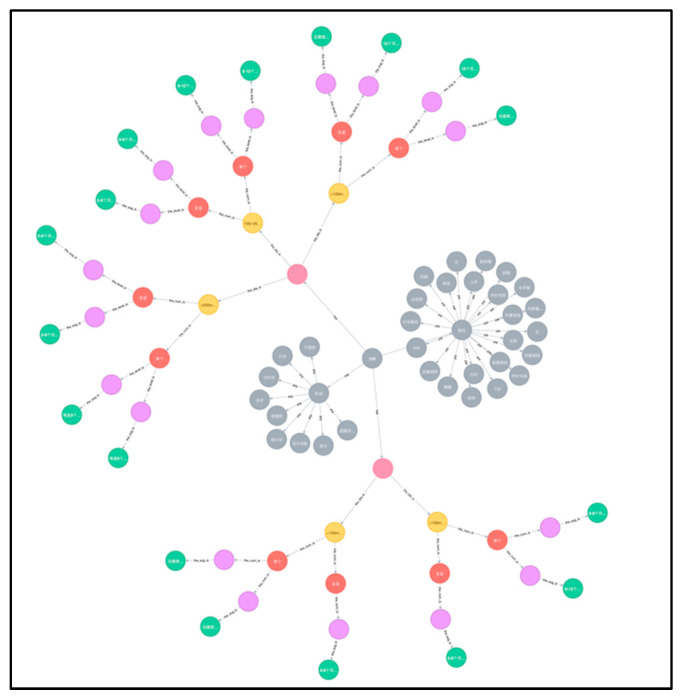
Partial display of knowledge graph for pulmonary nodule follow-up recommendations.

A graph database for pulmonary nodule follow-up recommendations was established as a small knowledge graph, based on multiple credible sources. The graph database contained six categories of entities, namely nodule name, size, quantity, solidity, risk level and follow-up recommendation of pulmonary nodule. It comprised a total of 168 different nodes. All cases of pulmonary nodules were included in sequential order along with a detailed follow-up recommendation for each specific nodule. The established knowledge graph is shown in Figure 3. Based on it, a more comprehensive knowledge graph for related lung diseases will be further explored and constructed.

In order to better illustrate the various types of entities in the graph knowledge database, we differentiate them by color as follows:

- The gray dots are used to determine the presence of nodules and their shape and location.
- The pink dots are used to describe the solidity or sub-solidity of the nodule.
- The yellow dots are used to describe the size of the nodule.
- The red dots are used to describe the number of nodules.
- The purple dots are used to describe the degree of risk of the nodule.
- The green dots are used to describe the follow-up recommendations for the nodules.

### 3.3. Demonstration of Entire System

Follow-up recommendations of the existing 48,091 (100%) reports were automatically generated based on the word segmentation results of NLP and the logical rules established by the knowledge graph. The number of results and their match percentages with the follow-up recommendations provided in the original reports are listed in Table 2. A total of 43,898 (91.28%) reports were found to contain follow-up recommendations that were written clearly by doctors and proven correct, indicating an accuracy of 91.28% compared to the follow-up recommendations generated by artificial intelligence. The number of reports recommending no required routine follow up was the largest (N = 37,049; 77.04%), and these reports exhibited the lowest match with the follow-up recommendations generated by the rule template. This indicates that doctors tend to skip explaining follow-up recommendations at this level when writing the report. In contrast, in reports that required a higher frequency of follow ups or more complicated follow-up methods, doctors rarely disregarded or misjudged the case.

**Figure 4:**
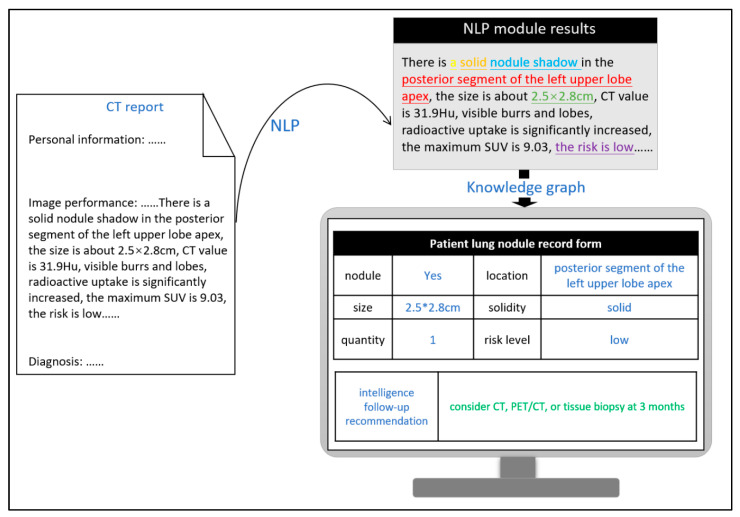
Demonstration of the intelligent generation process of follow-up recommendations for reports on pulmonary nodules.

Figure 4 depicts the actual process implemented in hospitals. After image descriptions of pulmonary nodules are processed, the preset entities are extracted using NLP techniques (the text presented in different colors in the figure denotes different types of entities) and subsequently documented in the patient’s record, which thus constitutes the attributed values of different variables in the query language of the knowledge graph. Finally, the system automatically generates follow-up recommendations by correlating these values and their relationships. By calculating the match between the system-generated follow-up recommendations and those provided in the radiologist reports, the quality management of follow-up recommendations is performed automatically. Furthermore, this process can be incorporated to provide near real-time suggestions to radiologists while writing the report with regard to follow-up recommendation quality control, thereby facilitating the generation of standardized follow-up recommendations at the writing stage. On average, it takes less than 0.5 s to process each report. Even when the response time spent on querying the knowledge graph and prompting the results is added to this processing time, the total time of realizing the function is negligible in comparison to the time spent writing the report.

## 4. Discussion

Radiology reports depict an objective reflection of the patient’s condition. They include the doctor’s comprehensive judgment and interpretation of the imaging results and convey the value of the field of image diagnostics. In terms of their functionality, they are an important tool for effective communication among radiologists, clinicians, and patients, which is essential for medical institutions to provide high-quality and efficient medical services. The free-text data form makes the reports more challenging for contextual and semantic analyses. Therefore, we explored the feasibility of performing upfront automatic quality control of radiology reports during the report writing stage using artificial intelligence.

The actual reports exhibit low-matching degrees with the obtained system results. This is potentially specific to China, where a hierarchical diagnosis and treatment system has not been strictly implemented. Namely, the clinician that treats the patient does not need to strictly follow the radiologists’ advice and retains significant freedom in the selection of medical imaging examinations. The implementation of a hierarchical diagnosis and treatment system in China [34] will hold frontline doctors to stricter guidelines to closely follow radiologists’ recommendations in their decision on whether to perform a specific medical imaging exam. Therefore, it is required that domestic radiologists prepare in advance by providing more accurate follow-up recommendations in their radiology reports and reduce the frequency of overuse, underuse and misuse of subsequent medical imaging exams.

From a technical perspective, our study shows that the NLP model resolves the unstructured data problem, which previously prevented the direct use of unstructured data on a large scale. The proposed model demonstrates both high accuracy and fast processing speed. The fast response ensures that the intelligent generation of follow-up recommendations does not increase the waiting time and workload for doctors. Therefore, the implementation of this function not only helps patients understand the report more clearly, but also helps doctors provide prompt recommendations. 

From a quality management standard perspective, the introduction of a knowledge graph in this study resolves the issue of inconsistent quality control standards due to individual and experience level differences among reviewing doctors. The established knowledge graph is based on several authoritative guidelines, making the quality management standards of the system reliable, objective and consistent. With regard to the quality management process, the proposed method enables quality management to be conducted in parallel with the writing of the report. In comparison to existing quality control methods, such as departmental spot checks after writing the reports, the upfront quality control of radiology reports aids radiologists in identifying non-standard writing and makes timely modifications based on radiology images. Thereby, the process reduces the occurrence of errors in the final report and enhances the report.

In this study, pulmonary nodule-related reports were selected as the subject of research mainly because of their clinical significance, as well as the comprehensiveness and availability of the associated guidelines. For patients with incidental pulmonary nodules, nodule detection is not among the main purposes of the patient’s medical visit or image examination. Although most pulmonary nodules are benign, there is still a risk of malignancy. Therefore, early detection and scientific assessment can save lives and substantially reduce medical costs. Moreover, listing detailed follow-up recommendations in the report can help patients gain trust in the medical system and build a closer relationship between the two entities while improving the efficiency and quality of the provided medical services, thereby raising the hospital’s value.

Nevertheless, problems regarding data structuring, mapping and knowledge graph construction require further in-depth research. Data structuring is an important aspect determining data quality. The development of a structured report template that meets the specific requirements of diagnosis and treatment in China can further improve the accuracy and efficiency of NLP. The mapping between Chinese medical oncology and English data remains to be addressed. A significant obstacle in the standardization of mapping and terminology has been the absence of a Chinese version of LABLEX, which was developed as a standardized radiology terminology compilation. Finally, in this study, the knowledge graph only focused on the quality control of pulmonary nodule follow-up recommendations, whereas the description of other pulmonary functions, such as pulmonary texture and fibrosis were omitted. Therefore, the establishment of extensive tailored knowledge graphs is essential for the application of this function in other clinical areas. Moreover, the quality of knowledge graphs should be validated based on three aspects including: (i) accuracy, (ii) consistency and (iii) conciseness [35]. This task was not implemented in this study and will be applied in the subsequent research involving the creation and verification of large-scale knowledge graphs.

## 5. Conclusions

In summary, a deep learning and CRF model was adopted to solve the problem of post-structured data of radiology reports. Subsequently, a knowledge graph was applied to build a connection between the NLP model results and rule templates to help solve quality management issues in radiology reports. Our results suggest that this method can be applied to pulmonary nodule reporting in real-world clinical practice, thereby achieving real-time quality control of pulmonary nodule follow-up recommendations.

## Figures and Tables

**Table 1:** Performance of natural language processing system.

Entity Type	Accuracy	Precision	Recall	F1	Number
Location	96.71%	96.00%	93.44%	94.70%	37
Shape	94.62%	96.70%	88.00%	92.15%	11
Nodule name	97.21%	98.95%	94.00%	96.41%	13
Solidity	93.13%	94.09%	94.47%	94.28%	7
Quantity	90.34%	89.34%	91.50%	90.41%	6
Risk level	89.34%	90.48%	90.20%	90.34%	14
Size	92.15%	93.08%	93.35%	93.21%	-
Follow-up recommendation	93.12%	96.64%	89.75%	93.07%	9
Total	94.22%	94.56%	93.96%	94.26%	97

**Table 2:** Number of reports and corresponding match percentages for different levels of automatically generated follow-up recommendations.

Follow-Up Recommendation Level	Number of Reports	Matching Rate
No routine follow up required	37,049	89.46%
CT review at 12 months	4501	95.02%
CT review between 6 and 12 months, and consider a subsequent CT review between 18 and 24 months	1539	98.38%
CT review between 3 and 6 months, and a subsequent CT review between 18 and 24 months	769	98.51%
Consider CT, PET/CT, or tissue biopsy at 3 months	1539	98.97%
CT review between 3 and 6 months, and if stable, consider subsequent CT reviews at 2 and 4 years	1731	99.87%
CT review between 6 and 12 months, and if nodule is persistent, subsequent CT review every 2 years within a 5-year period	500	99.17%
CT review between 3 and 6 months, and if nodule is persistent or solid content < 6 mm, subsequent CT review every year within a 5-year period	269	98.77%
CT review between 3 and 6 months, and then consider subsequent CT reviews according to status of most suspicious nodule	192	98.25%
Total	48,091	91.28%

## Data Availability

The datasets generated and/or analyzed during the current study are not publicly available due to research progress issues but are available from the corresponding author on reasonable request.

## Abbreviations

ACR	American College of Radiology
Lung-RADS	lung imaging reporting and data system
NLP	Natural Language Processing
CRF	Conditional Random Field
Bi-LSTM	Bi-directional long short-term memory
